# Assessing the influence of the rhizosphere on soil hydraulic properties using X-ray computed tomography and numerical modelling

**DOI:** 10.1093/jxb/eru509

**Published:** 2015-03-04

**Authors:** Keith R. Daly, Sacha J. Mooney, Malcolm J. Bennett, Neil M. J. Crout, Tiina Roose, Saoirse R. Tracy

**Affiliations:** ^1^Bioengineering Sciences Research Group, Faculty of Engineering and Environment, University of Southampton, University Road, Southampton SO17 1BJ, UK; ^2^School of Biosciences, University of Nottingham, Sutton Bonington Campus, Leicestershire LE12 5RD, UK

**Keywords:** Bulk soil, image-based homogenization, matric potential, rhizosphere, soil pores, water release characteristic, X-ray computed tomography.

## Abstract

Understanding the dynamics of water distribution in soil is crucial for enhancing our knowledge of managing soil and water resources. The application of X-ray computed tomography (CT) to the plant and soil sciences is now well established. However, few studies have utilized the technique for visualizing water in soil pore spaces. Here this method is utilized to visualize the water in soil *in situ* and in three-dimensions at successive reductive matric potentials in bulk and rhizosphere soil. The measurements are combined with numerical modelling to determine the unsaturated hydraulic conductivity, providing a complete picture of the hydraulic properties of the soil. The technique was performed on soil cores that were sampled adjacent to established roots (rhizosphere soil) and from soil that had not been influenced by roots (bulk soil). A water release curve was obtained for the different soil types using measurements of their pore geometries derived from CT imaging and verified using conventional methods, such as pressure plates. The water, soil, and air phases from the images were segmented and quantified using image analysis. The water release characteristics obtained for the contrasting soils showed clear differences in hydraulic properties between rhizosphere and bulk soil, especially in clay soil. The data suggest that soils influenced by roots (rhizosphere soil) are less porous due to increased aggregation when compared with bulk soil. The information and insights obtained on the hydraulic properties of rhizosphere and bulk soil will enhance our understanding of rhizosphere biophysics and improve current water uptake models.

## Highlight

Using non-destructive imaging techniques and numerical modelling, we quantify differences in hydraulic and structural properties of bulk and rhizosphere soil for sand and clay loam soils.

## Introduction

The concept of the ‘rhizosphere’, proposed by Hiltner (1904), refers to the volume of soil adjacent to a plant root over which the root has influence. The rhizosphere is created from root–soil–microbe interactions and the compression of soil due to root expansion (Dexter, 1987; Whalley *et al.*, 2005; Aravena *et al*., 2011, 2014). Soil physical structure affects root growth; however, in turn, a growing root physically alters the soil structure through the creation of biopores (Stirzaker *et al.*, 1996), which impact on fluid transport through soil (Angers and Caron, 1998). Root water uptake leads to further soil structural changes through drying which may cause soil shrinkage (Towner and Childs, 1972). The root also secretes chemical compounds, referred to as exudates, into the surrounding soil. These exudates can be divided into three categories: (i) mucilage, which is usually found at the root tips and consists of polysaccharides and uronic acids; (ii) molecules excreted by the root hairs such as amino acids, organic acids, and simple sugars; and (iii) cellular organic substances produced by root epidermis senescence (Tan, 2000). Gases, including carbon dioxide and methane, are also released from roots, although some researchers (Swinnen *et al.*, 1995; Grayston *et al.*, 1997) do not define them as exudates as they diffuse into the atmosphere. Aside from the gases released by roots, the remaining exudates constitute a resource that is highly valued by microorganisms, resulting in a much greater diversity of microorganisms in the rhizosphere than in the surrounding bulk soil (Smalla *et al.*, 2001). The microbial community that exists in the rhizosphere results in several dynamic processes, some of which aid nutrient cycling and aggregation of soil particles. The release of root exudates into the soil also changes its chemical and physical characteristics, which enhances microbial growth (Gregory, 2006).

Soil characteristics within the rhizosphere are thought to be markedly different from those of the bulk soil. For example, rhizosphere soil has been shown to contain greater numbers of the largest pore sizes (Whalley *et al.*, 2005) and is generally more acidic than bulk soil, with denitrification being more rapid (Tan, 2000). The hydraulic properties of rhizosphere soil are hypothesized to differ from those of bulk soil; for example, some root exudates cause hydrophobicity of soil particles which affects their wetting ability (Czarnes *et al.*, 2000). In addition, root exudates act like glue by aiding the aggregation of soil particles in the rhizosphere, while also decreasing the wetting rate (Czarnes *et al.*, 2000; Hallett *et al.*, 2009). This stabilizing effect is enhanced in dry soil in which the viscosity of root exudates is increased (Walker *et al.*, 2003). Root exudates are also important in maintaining root–soil contact in drying soils. As the soil dries, the surface tension of the exudate decreases, increasing its ability to wet surrounding soil particles (Read and Gregory, 1997). Other studies suggest that rhizosphere soil may be wetter than bulk soil (Young, 1995) due to the formation of a coherent sheath of soil permeated by mucilage and root hairs, known as the rhizosheath (Gregory, 2006). Small quantities of water are released from the root to the rhizosheath at night, while the root absorbs water from the rhizosheath during the day (Walker *et al.*, 2003). The rhizosheath therefore has a significant effect on soil hydraulic properties, and roots in general modify the soil structure, affecting the water retention capacity of soil.

In order to investigate the above effects on the hydraulic properties of soil, non-invasive measurements of soil structure are required. There has been significant growth in the use of X-ray computed tomography (CT) as a method to visualize and quantify water flow in soil non-destructively (Crestana *et al.*, 1985; Mooney, 2002; Mooney *et al.*, 2012). Mathematical modelling combined with CT has also been widely used to obtain properties of porous materials based on pore scale geometries (Blunt *et al.*, 2013), and to understand the effect of root-induced compaction using a Darcy–Richards’ formulation (Aravena *et al*., 2011, 2014). Recently, Tracy *et al.* (2015) combined CT imaging and image-based quantification with numerical modelling (Pavliotis and Stuart, 2008; Daly and Roose, 2014) to calculate the hydraulic conductivity of soil using direct measurements of soil pore structure under a range of different saturation conditions.

Here the application of this method to quantify water distribution in soil pores for bulk and rhizosphere soil in contrasting soil textures is demonstrated. By combining CT imaging with mathematical modelling and up-scaling techniques, it is possible to determine the effect of a living root system on shaping the soil structure (i.e. rhizosphere morphology) on the hydraulic and structural properties of soil under a range of different saturation conditions.

## Materials and methods

### Sample preparation

Soil was obtained from the University of Nottingham farm at Bunny, Nottinghamshire, UK (52.52°N, 1.07°W). The soils used in this study were a Eutric Cambisol (Newport series, loamy sand/sandy loam) and an Argillic Pelosol (Worcester series, clay loam). Particle size analysis for the two soils was: 83% sand, 13% clay, and 4% silt for the Newport series; and 36% sand, 33% clay, and 31% silt for the Worcester series. Typical organic matter contents were 2.3% for the Newport series and 5.5% for the Worcester series (Mooney and Morris, 2008). Loose soil was collected from each site in sample bags, sieved to <2mm, and packed into columns (120mm height, 60mm diameter) at a bulk density of 1.2 Mg m^–3^. The soil was mixed to distribute the different sized soil particles evenly before pouring it in small quantities into the columns. After compacting each layer, the surface was lightly scarified to ensure homogeneous packing and hydraulic continuity within the column (Lewis and Sjostrom, 2010). The soil columns were saturated slowly by wetting from the base for 12h and allowed to drain freely for 48h. All columns were weighed and maintained at this weight throughout the experiment by adding the required volume of water daily to the top of the column to ensure soil moisture content remained near a notional field capacity. Half the columns were planted with a single wheat seed (cv. Zebedee) and grown for 4 weeks in a growth room, 16h day/8h night, day temperature 24 ºC, night temperature 18 ºC, 50% humidity. At the end of the growth period, small soil cores (10mm height, 10mm diameter) were carefully excavated from the centre of the soil columns. The columns that contained a plant were considered to have developed a rhizosphere, while those without were considered to contain only bulk soil. The samples were then CT scanned (below). Saturated hydraulic conductivity measurements of all cores were obtained using a constant head device (Rowell, 1994), for comparison with the model-derived values.

### Soil water release characteristic (WRC)

A custom-built vacuum chamber was designed in order to hold the soil sample at a given matric potential whilet undergoing CT scanning as outlined in Tracy *et al.* (2015). The chamber contained a porous ceramic plate (Soil Moisture Corp, Santa Barbara, CA, USA) on top of which a soil core was placed, with kaolin clay at the base to ensure a good contact. The porous ceramic was first submerged in de-aerated water and a vacuum applied to ensure no air bubbles remained trapped within the ceramic. A 0387 Millipore vacuum pump (Merck Millipore, MA, USA) was attached to the chamber and the soil cores were initially saturated before being put under successive vacuums of –5, –10, –20, –40, –60, and –75 kPa. The vacuum pump was turned on for 120min then the valve sealed to retain the vacuum inside the chamber. At each successive matric potential the soil core inside the chamber was scanned. After each scan, the soil core was removed from the chamber and weighed to calculate water content.

To obtain a conventional WRC for both soils, a pressure plate Model 1600 Pressure Plate Extractor (Soil Moisture Corp) was used. The soil core samples were placed on the plate and weighed frequently until equilibrated at each matric potential. After the final measurement, the samples were oven dried at 105 °C for 24h then weighed.

### X-ray computed tomography

Three replicate cores from each treatment (bulk or rhizosphere soil) and soil type (sand or clay) of the cores were scanned at the seven matric potentials (0 to –75 kPa) giving a total of 84 scans. X-ray CT scanning was performed using a Phoenix Nanotom 180NF (GE Sensing & Inspection Technologies GmbH, Wunstorf, Germany). The scanner consisted of a 180kV nanofocus X-ray tube fitted with a diamond transmission target and a five megapixel (2316×2316 pixels) flat panel detector (Hamamatsu Photonics KK, Shizuoka, Japan). A maximum X-ray energy of 100kV and 140 μA was used to scan each soil core. A total of 1440 projection images were acquired over a 360° rotation. Each projection was the average of three images acquired with a detector exposure time of 1 s. The resulting isotropic voxel edge length was 10.17 μm and total scan time was 105min per core. Although much faster scan times are possible, it was necessary in this instance to use a longer scan time to acquire the highest quality images to aid with the phase separation of the different soil constituents. Two small aluminium and copper reference objects (<1mm^2^) were attached to the side of the soil core to assist with image calibration and alignment during image analysis. Reconstruction of the projection images to produce three-dimensional (3D) volumetric data sets was performed using the software datos|rec (GE Sensing & Inspection Technologies GmbH).

The reconstructed CT volumes were visualized and quantified using VG StudioMAX^®^ 2.2 (Volume Graphics GmbH, Heidelberg, Germany). Air, soil, and water phases of the scanned volumes were segmented using a threshold technique based on measurements from two reference objects, which were included in each scan; one contained a soil pore water sample and the other finely sieved soil (<100 μm). The definition of the phases was based on their differences in X-ray attenuation which are represented as greyscale values in the reconstructed CT volumes. This process is described further in Tracy *et al.* (2015). Image stacks of the extracted volumes for each phase were exported and subsequently analysed for individual pore characteristics using ImageJ v1.42 (http://rsbweb.nih.gov/ij/docs/user-guide.pdf) (Ferreira and Rasband, 2011). For 2D analysis, objects less than two pixels (twice the resolution) in diameter (0.02mm) and for 3D analysis objects less than two voxels in each direction (8×10^–6^ mm^3^) were considered as potential noise as a precaution (Wildenschild *et al.*, 2005), and subsequently excluded from the analysis.

**Fig. 1.**
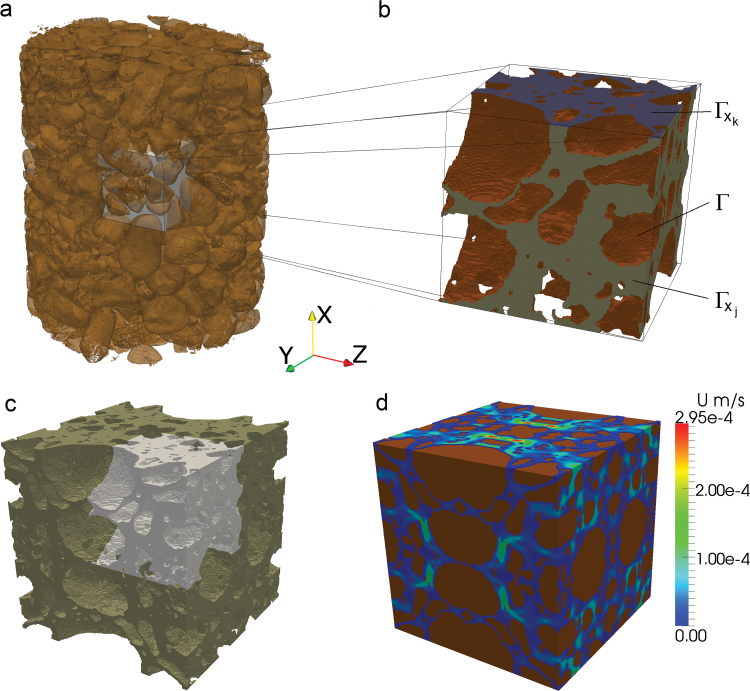
Schematic showing (a) subsampling of segmented volume, (b) subsampled geometry with boundaries Γ*x*_*k*_, Γ*x*_*j*_, and Γ for *k*=1, (c) the resulting truly periodic geometry created by reflection of the subsampled region in the *x*-, *y*-, and *z*-axis, and (d) a typical solution to the cell problem showing the absolute velocity. (This figure is available in colour at *JXB* online.)

In order for the geometries of the water-filled pores (WFPs) to be modelled, surface mesh files (.stl) were required; which were generated in VG StudioMax v2.2. After segmentation of the soil water phase, a cube-shaped region of interest (ROI) template was imported. Each sample was subsampled, from random initial co-ordinates, with six cubes comprising side lengths of 3.8mm, giving an overall cube volume of *V*_m_=54.9mm^3^ (Fig. 1). The same co-ordinates were used for different matric potentials of the same sample.

### Numerical modelling

To understand the differences between the properties of the rhizosphere and bulk soils, the hydraulic conductivity is calculated using the method of homogenization (Pavliotis and Stuart, 2008). This technique enables Darcy’s law to be derived from Stokes’ equations for fluid flow and, through a mathematically rigorous up-scaling, the hydraulic conductivity to be calculated based on a representative elementary volume (REV). Full details of the scaling and resulting equations can be found in Daly and Roose (2014) and Hornung (1997). Further discussion of the assumptions used and their applicability in this context are described in Tracy *et al.* (2015). Here the underlying assumptions, the method, and resulting equations are summarized.

There are several key assumptions that are made in order to develop the model. First it is observed that for typical pore sizes the viscous forces dominate the flow properties (Fowler, 1997). Hence, the Stokes limit of the Navier–Stokes equations where all inertial terms are neglected may be considered. Secondly, it is required that the soil structure is periodic (i.e. it is made up of regularly repeating units and, hence, a single one of these units is representative of the overall soil properties). Clearly for real soil samples this is not the case, and an apparent, image-based, periodicity is enforced by reflection of the REV (Fig. 1). The error induced by enforcing periodicity is that the geometry considered numerically is now fully periodic rather than quasi-periodic and does not truly represent the imaged soil structure. To overcome this, different size REVs were taken from the segmented *.stl files.

The REVs sampled from the six cubes were of volume, *V*=*V*_m_/2_*j*_, where *j* is a positive integer in the range 0–8 such that the smallest volume considered is 0.2mm^3^ and the largest is *V*_m_. As *j* is decreased and, hence, the size of the REV is increased, the relative size of the errors induced by the reflection decreases. Similarly as the REV size increases, the hydraulic properties of the subsample will, in principle, converge to the hydraulic properties of the soil. Finally, as it is possible to segment the air and water separately from the CT scan image of the soil structure, the fluid dynamics can be greatly simplified. Rather than consider the moving interface between each phase, the relatively slow flow of water about a fixed interface is considered. The equations are further simplified by assuming that the non-wetting phase, in this case air, is stationary. If this is not the case, then the movement of the air effectively lubricates the movement of water, resulting in an increase in the hydraulic conductivity. This approach is valid assuming first that the pressure gradients are sufficiently low that the interface remains fixed and secondly that the non-wetting phase is not connected and, hence, the trapped non-wetting phase has zero average velocity.

After a rigorous mathematical analysis of Stokes equations, it was possible to derive Darcy’s law which is valid for the bulk or rhizosphere soil and describes fluid driven by an external pressure gradient (see Hornung, 1997; Daly and Roose, 2014;). The average water velocity *u* is given by

$$u=-K\left(\nabla p_{0}-\rho g{\hat{e}}_{z}\right),$$ (1)

where ρ is the fluid density ($\rho =10^{3}{\text{ kg m}}^{-3}$ in the case of water), $g=9.8\;{\text{m s}}^{-2}$ is the acceleration due to gravity, $p_{0}$ is the applied pressure, and K  is the relative permeability (in the general case a tensor) which has components defined as

Kjk=Ly2μ∫Ωwe^j⋅νk dy. (2)

Here, ${\hat{e}}_{j}$ for j=x,y,z is a unit vector in the *j* -th direction, μ is the viscosity ($\mu =10^{-3}{\text{ kg m}}^{-1}\;s^{-1}\;$ in the case of water), $L_{y}$ is the length of the REV, and $\nu {\;}_{k}^{}$ is the local velocity. The hydraulic conductivity is defined as the average water velocity driven by gravity. Assuming that the air velocity is slower than the water velocity, local ‘corrector’ velocity $\nu {\;}_{k}^{}$ satisfies the following set of equations which are solved on a single REV a single time to parameterize Equation 2,

$$\nabla^{2}\nu_{k}-\nabla \pi_{k}={\hat{e}}_{k},\;\;\nabla \cdot \nu_{k}=0,\;x\in \Omega_{\;w},$$ (3a)

$$\nu_{k}=0,\;x\in \Gamma ,$$ (3b)

$$\pi_{k}=0,\;\frac{\partial }{\partial x_{k}}\left({\hat{e}}_{k}\cdot v_{k}\right)=0,\;{\hat{e}}_{j}\cdot v_{k}=0,\;j\neq k,\;x\in \Gamma x_{k},$$ (3c)

$$\frac{\partial \pi_{k}}{\partial x_{j}}=0,\;\frac{\partial }{\partial x_{p}}\left({\hat{e}}_{p}\cdot v_{k}\right)=0,\;{\hat{e}}_{j}\cdot v_{k}=0,\;p\neq k,\;p\neq j,\;x\in \Gamma x_{j},$$ (3d)

where $\pi_{k}$ is the local pressure correction due to the microscale geometry, $\Omega_{\;w}$ is the water domain, $\Gamma x_{k}$ is the boundary located at $x_{k}=0$ , $x_{k}=1/2$ , $\Gamma x_{j}$ is the union of the boundaries located at $x_{j}=0$ , and $x_{j}=1/2$ for j≠k , and Γ is the union of the soil–water interface and the air–water interface (Fig. 1). Physically this problem in Equations 3a–3d can be thought of as calculating the fluid velocity subject to a unit pressure gradient in the direction of ${\hat{e}}_{k}$ . As the equations are linear, Darcy’s law follows by multiplying the resulting solution by the pressure gradient.

Equations 3 were solved numerically on each subsample obtained from the CT images. The equations were solved using OpenFOAM, an open source Computational Fluid Dynamics toolbox running on IRIDIS, the High Performance Computing Facility at the University of Southampton. The result is a set of hydraulic conductivity calculations that converge to the true hydraulic conductivity of the soil, at each point along the WRC, as the subvolume size is increased.

To quantify the results further, the van Genuchten model for the WRC and the unsaturated hydraulic conductivity (van Genuchten, 1980) was fitted to the calculated values using a non-linear least squares method. The volumetric water content θ is given by

$$\theta =(\theta_{s}-\theta_{r}){\left(\frac{1}{1+{\left(\alpha h\right)}^{n}}\right)}^{m}+\theta_{r},$$ (4)

where $\theta_{s}$ and $\theta_{r}$ are the saturated and residual volumetric water content respectively, *h* is the hydraulic head, m=1−1/n , and *n* and α are the van Genuchten parameters. The corresponding hydraulic conductivity is given by $K=K_{sat}k_{r}^{vg}$ . Here $K_{sat}$ is the saturated hydraulic conductivity, and the relative hydraulic conductivity is given by

$$k_{r}^{vg}=\frac{{\left\{1-{\left(\alpha h\right)}^{n-1}{\left[1+{\left(\alpha h\right)}^{n}\right]}^{-m}\right\}}^{2}}{{\left[1+{\left(\alpha h\right)}^{n}\right]}^{m/2}}.$$ (5)

$\theta_{r}$ is taken to be negligible and the remaining parameters were fit to the imaged data.

### Statistical analysis

The results obtained directly from the CT images were analysed by general analysis of variance (ANOVA) containing soil type and matric potential and all possible interactions as explanatory variables using Genstat 15.1 (VSN International, UK). The probability of significance *P*, with a threshold value of (*P*<0.05), corresponding to a 95% confidence limit, was calculated and is used as a measure of significance of the results obtained.

## Results and Discussion

### Hydraulic properties

**Fig. 2.**
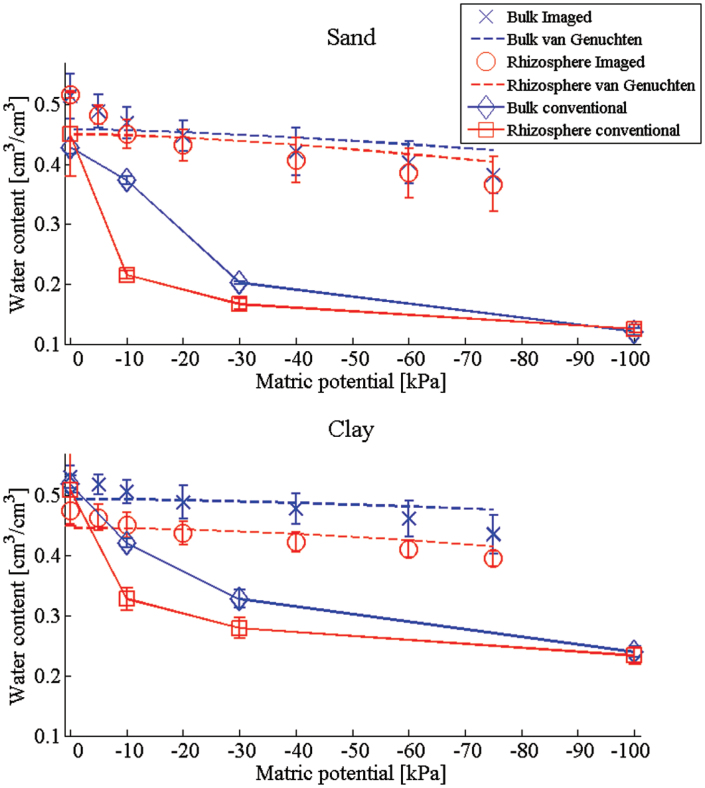
Water release characteristic of the sand and clay bulk and rhizosphere soils for the conventional and imaging methods. (This figure is available in colour at *JXB* online.)

The WRC was obtained via conventional methods and the imaging method (Fig. 2) for bulk and rhizosphere soil in the two soil textures. Despite the differences between the methods, the image-based approach does capture the differences between the bulk and rhizosphere soils. For both soil types, more water is retained in the bulk soil than within the rhizosphere (Fig. 2). Measured in the conventional way, this trend is observable for both the sand and clay soils. However, using the imaging method, only the clay soil shows a significant difference between the bulk and rhizosphere soils. In general, the imaging method provides a good estimate of the volumetric water content at 0 kPa. The method performs less well and provides a noticeable overestimate at more negative matric potentials, compared with the conventional method. The result is that the slope of the WRC with matric potential, which is a key parameter in Darcy–Richards’ flow models (Hornung, 1997), is underestimated.

From the conventionally measured WRC, it can be seen that the bulk clay soil responded the least to a decrease in matric potential (Fig. 2). The volumetric water content at saturation was high and the soil retained the majority of this water across the matric potential range. The rhizosphere clay soil behaved similarly to the bulk clay soil. However, the initial drainage of the soil from saturation to –30 kPa was much steeper and the resulting volumetric water content was lower compared with the bulk clay soil. The sand soils drained to lower volumetric water contents compared with the clay soils (Fig. 2). The rhizosphere sand responded strongly to the decreased matric potential, losing almost half of its water content by –30 kPa. The bulk sand showed an initial lag in drainage; however, by –30 kPa, the volumetric water content was similar to that of rhizosphere sand. It would appear that the bulk sand soil required a slightly lower matric potential (more than –10 kPa) for drainage to occur compared with rhizosphere sand. The differences observed in the WRC between the bulk and rhizosphere soils were most significant for matric potentials less than –10 kPa for the sand soil and –20 kPa for the clay soil. Hence, there is a significant difference between the behaviour of the different soil types at –30 kPa, a typical field capacity (Richards and Weaver, 1944). These trends are also observed in the imaged data (Fig. 2), although the differences between the different soils are less significant.

**Fig. 3.**
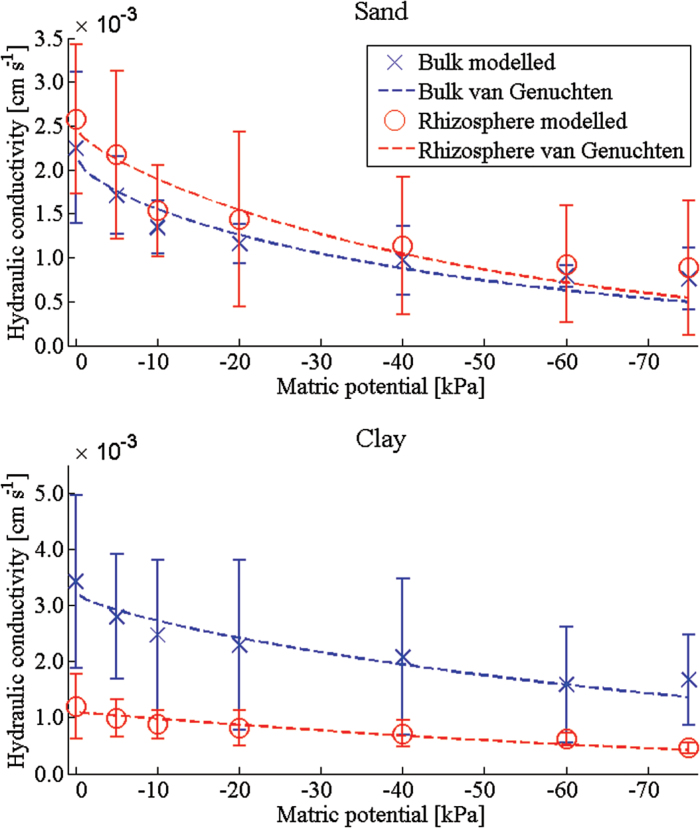
Calculated hydraulic conductivity values for clay and sand soils. Data are plotted for bulk and rhizosphere soils, and a van Genuchten curve has been fitted through these data using a non-linear least squares method. The parameters are given in Table 1. (This figure is available in colour at *JXB* online.)

**Fig. 4.**
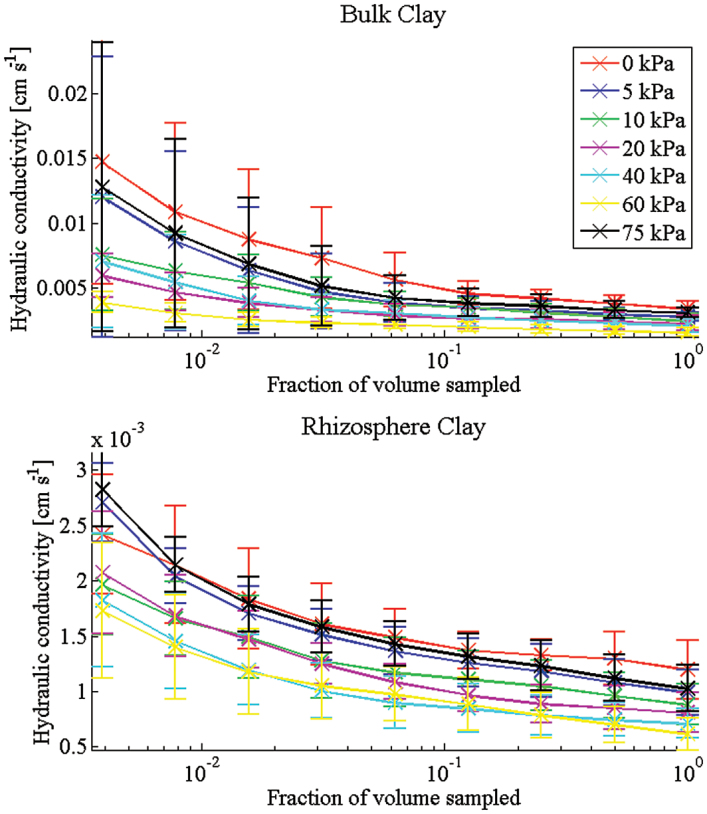
Convergence plots for clay soil. For each case (rhizosphere and bulk), three samples were taken. From each of these, six subsamples were obtained. These plots show the average and standard deviation over the 18 subsamples for increasing subsample size. (This figure is available in colour at *JXB* online.)

**Fig. 5.**
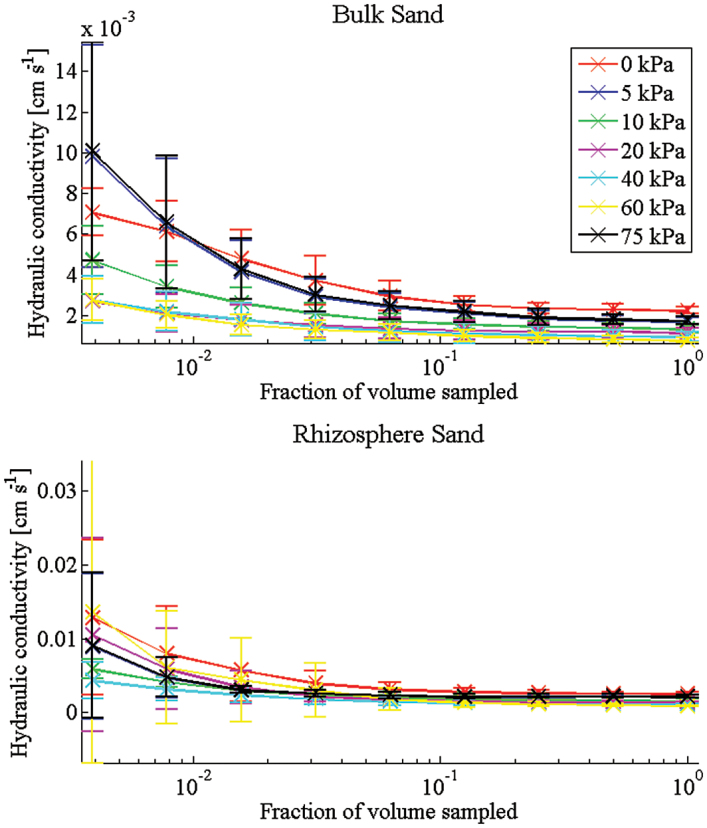
Convergence plots for sand soil. For each case (rhizosphere and bulk), three samples were taken. From each of these, six subsamples were obtained. These plots show the average and standard deviation over the 18 subsamples for increasing subsample size. (This figure is available in colour at *JXB* online.)

**Fig. 6.**
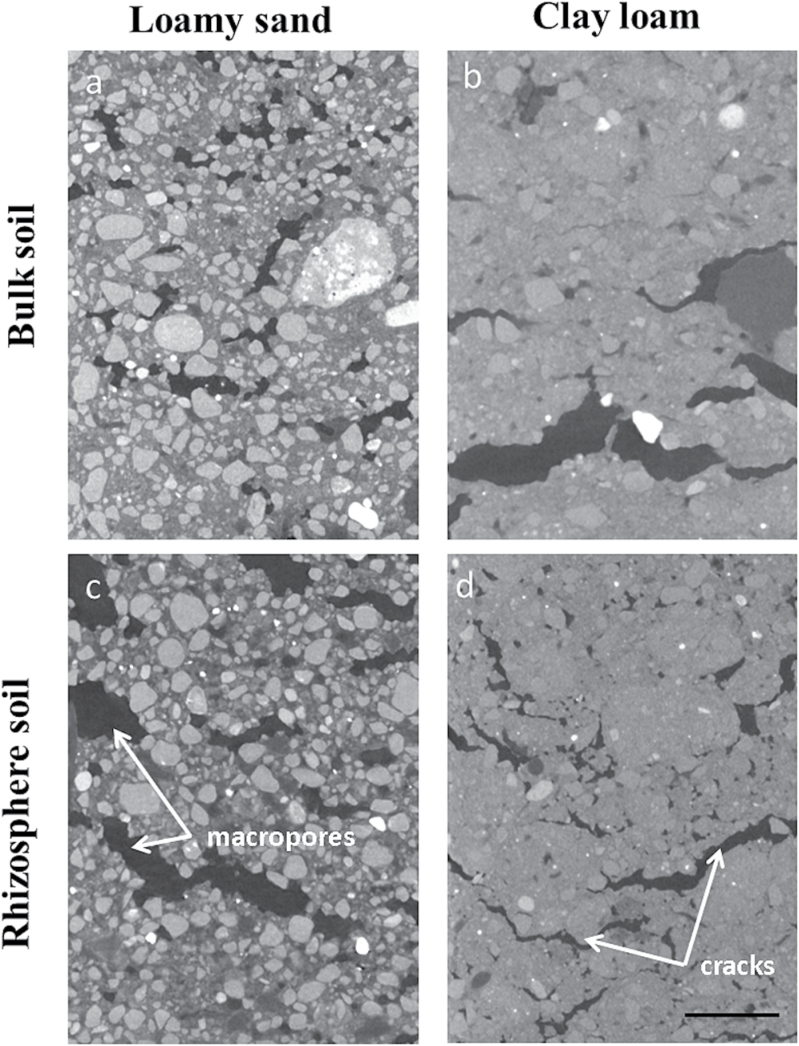
Greyscale images of bulk sand (a), clay (b) and rhizosphere sand (c), and clay (d) soils. Annotations highlight the presence of macropores in sand soil and crack formation in the clay. Scale bar=2.5mm. (This figure is available in colour at *JXB* online.)

**Fig. 7.**
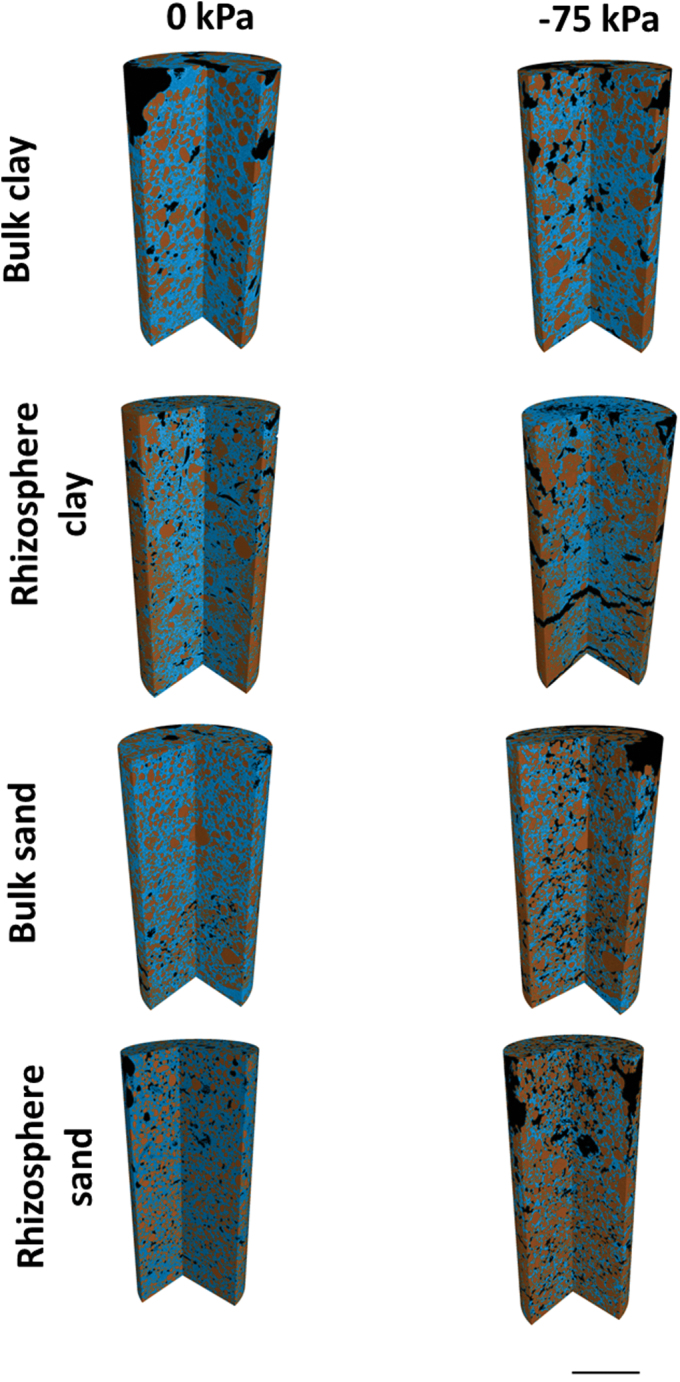
3D core sections of sand and clay, bulk and rhizosphere soil samples at the matric potentials 0 and –75 kPa. Segmented phases are soil, water-filled pores, and air-filled pores. Scale bar=5mm. (This figure is available in colour at *JXB* online.)

The trends in the WRC are supported in the hydraulic conductivity predictions (Fig. 3). For all soils, the value of the hydraulic conductivity is seen to converge approximately to a fixed value as the REV size is increased (Figs 4, 5). It should be noted that the negative values obtained for low REV size in Fig. 5 do not correspond to a negative hydraulic conductivity. Rather these values tell us that with a REV this small the average hydraulic conductivity is smaller than the standard deviation and there is no correlation between the values obtained. As the size of the REV is increased, the correlation increases and all values become positive. The predicted hydraulic conductivity values are seen to compare with reasonable accuracy with the measured value at 0 kPa (Table 1). Here there is a significant difference observed between the bulk and rhizosphere hydraulic conductivities for the clay soil and relatively little difference for the sand soil. The sizeable error bars in these figures are attributed to natural variation in the soil samples that can occur even in repacked soil samples. Despite these variations, it is clear that there is a measurable difference between the calculated hydraulic conductivity of the bulk and rhizosphere soils. The calculated hydraulic conductivity for the bulk clay soil is quite high and corresponds to a high number of macropores and cracks (Figs. 6, 7). It is here that the differences in bulk and rhizosphere soil can be most clearly observed as the rhizosphere clay soil has the lowest hydraulic conductivity of the soils considered. In the clay soil, a bimodal distribution of pores was observed after successive wetting and drying cycles (Peng *et al.*, 2007); the pore sizes consist of a large number of subresolution micropores and a smaller number of large cracks and macropores (Fig. 6). The large reduction in hydraulic conductivity seen in the clay soil is related to a reduction in the diameter of the pores which contribute significantly to the hydraulic conductivity as the soil drains. This supports the hypothesis that one of the main effects of root exudates is to aid aggregation, reducing the overall macroporosity. In the case of the sand soil, there is a wider range of pore sizes. Hence, the root system has a significantly smaller effect on the overall soil pore size range. In summary, the macroporosity may decrease but, due to the wider range of pore sizes, this has less effect on the overall hydraulic conductivity. Alternatively, the main differences in soil structure may be occurring below the resolution of the CT images.

**Table 1.** Calculated and measured saturated hydraulic conductivity values and the van Genuchten parameters used to fit the calculated data

Soil	Measured *K*_sat_ (cm s^–1^)	Calculated *K*_sat_ (cm s^–1^)	Saturated volumetric water content θ_s_	α (cm^–1^)	*N*
Bulk sand	0.00225	0.00215	0.458	0.052	1.65
Rhizosphere sand	0.00276	0.00246	0.450	0.064	1.77
Bulk clay	0.00208	0.00321	0.494	0.032	1.75
Rhizosphere clay	0.00136	0.00109	0.446	0.051	1.98

The van Genuchten curves were fitted to the calculated hydraulic conductivity (Fig. 3) and the imaged WRC (Fig. 2). The resulting parameter values are given in Table 1. The curves are seen to fit the hydraulic conductivity well for all cases. The WRC fit is less favourable, with the slope of the van Genuchten curves for both the sand and clay being underpredicted. In the case of the clay soil, the comparison is reasonable with a slight underprediction of the volumetric water content at low matric potentials. However, in the case of the sand soil, the fit is less good. This suggests that there may be significant subresolution processes occurring which it was not possible to detect.

The results indicate that sand soil responded to the change in pore water pressure more than the associated clay soil, leading to a reduced volumetric water content compared with clay soil. While the differences were not as great as expected, this trend could be predicted due to the dominant particle size for the respective soils (i.e. the water in the clay soil is retained in the predominantly smaller pores). The clearest difference observed from the WRC, measured in the conventional way, was the variation in drainage between the bulk and rhizosphere soils. The presence of a higher percentage of clay in the clay soil meant that the soil structure is more prone to structural change, for example shrinkage, as the soil drained. Hence, the reason for the greater difference in the clay soil between the bulk and rhizosphere soil may be that the additions of root exudates and possible enhanced microbial activity in the rhizosphere soil intensified the aggregate formation process (Helliwell *et al.*, 2014). This effect may not have been seen as strongly in the sand soil, as this soil only had an average clay content of 13% and previous research suggests that a >12% clay content is required for aggregate formation in natural soils (Horn and Smucker, 2005). This result highlights that any ‘rhizosphere effect’ may be exhibited more strongly in soils with a high clay content and illustrates the requirement for studies that utilize contrasting soil textures as the majority of previous bulk and rhizosphere soil research focused on a single soil texture (Czarnes *et al.*, 2000; Smalla *et al.*, 2001; Whalley *et al.*, 2005). As the clay soil exhibited large-scale changes in both porosity and volumetric water content, there must be significant large-scale structural changes occurring brought about by the rhizosphere. The data suggest that, in the clay soil, the main effect of the root is to reduce the porosity through densification (Dexter, 1988) (Fig. 6) and decrease the rate of drainage (Fig. 2). In the sand soil, the main observed difference is an increase in drainage (Fig. 2), with little observable effect on the hydraulic conductivity. This suggests that, in addition to the increased aggregation in the clay soil, additional effects are occurring in the rhizosphere to alter the ability of the soil to retain water.

### Soil pore characteristics

In order to quantify the global air and water content per sample by imaging, air-filled pores (AFPs) and WFPs are defined as single connected regions of air or water, respectively. The pore space is also defined as the union of all the AFPs and WFPs. In addition, individual pores within the soil are referred to as simple connected pathways between two distinct points within the pore space. Typically, the pore space contained a single large WFP that contains >50% of the water within the pore space and a large number of much smaller AFPs and WFPs. The connected WFPs are the main contributor to both the WRC and the hydraulic conductivity calculations, and the WFP volume is analogous to the volumetric water content (Fig, 2). However, further insight may be gained into the wetting and drying behaviour of the soils by considering the properties of the AFPs and the total WFP surface area.

**Fig. 8.**
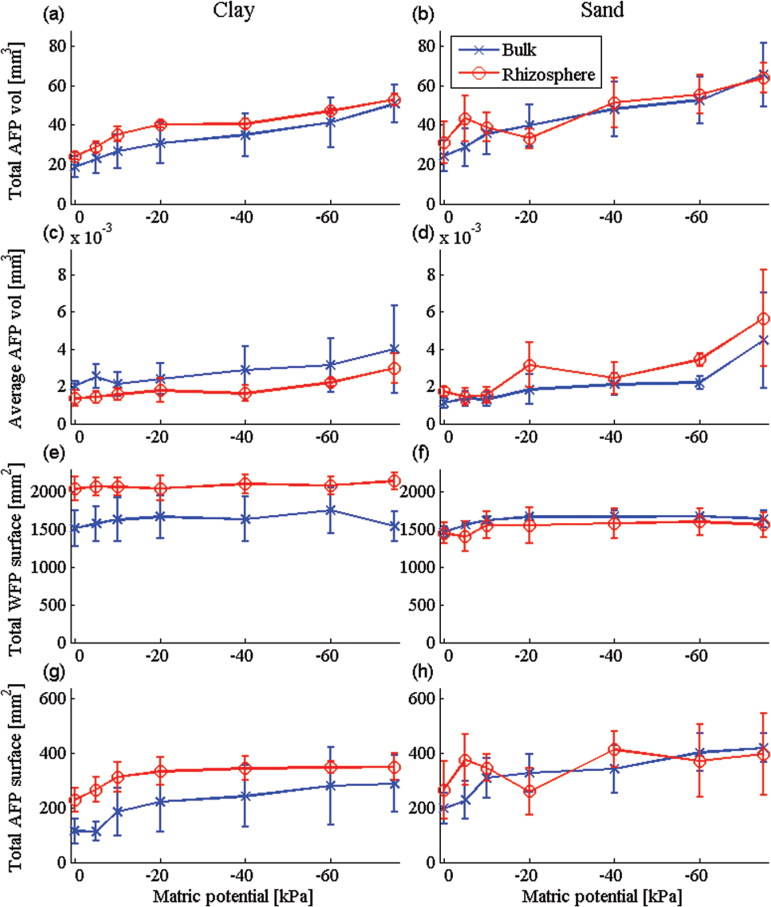
Total AFP volume for clay (a) and sand (b) soil, average AFP volume for clay (c) and sand (d) soil, total WFP surface area for clay (e) and sand (f) soil, and total AFP surface area values for clay (g) and sand (h) soil at the specific matric potentials. Error bars associated with histograms show one standard deviation. (This figure is available in colour at *JXB* online.)

The water-filled porosity decreased with decreasing matric potential (Figs 2, 7; *P*<0.001). There were no significant differences between total WFP in bulk and rhizosphere soil for both soil types. Previous work by Whalley *et al.* (2005) found that bulk and rhizosphere soils had similar porosities, but contrasting structures, which altered the water retention characteristics. The overall proportion of WFP space reduced by a total of 14% in bulk clay, 26% in bulk sand, 16% in rhizosphere clay, and 30% in rhizosphere sand soil from 0 to –75 kPa. The total volume of AFP space increased significantly (Figs. 7, 8; *P*<0.001) with decreasing matric potential from saturation (0 kPa). The rhizosphere soil contained larger quantities of AFPs (82.3mm^3^) compared with bulk soil (69.5mm^3^), but the difference was not significant. At 0 kPa the average AFP volume was 45mm^3^ for clay and 51mm^3^ for sand; this increased to just 87mm^3^ in clay and 101mm^3^ in sand (Fig. 8; *P*<0.001). There were no significant differences between the average volumes of the individual AFPs at the different matric potentials or soil types.

The total surface area of the WFPs generally increased as the matric potential decreased (Fig. 8; *P*<0.001). This trend was observed for all treatments. Rhizosphere soil had a greater total WFP surface area (1804mm^2^) compared with bulk soil (1616mm^2^), although the difference was not significant. The total WFP surface area was 1618mm^2^ in bulk clay and 2079mm^2^ in rhizosphere clay, 1615mm^2^ in bulk sand, and 1529mm^2^ in rhizosphere sand. Although the total volume of WFPs decreased as matric potential decreased (Fig. 2), the surface area increased across successive draining (until –60 kPa). Hence, as the size of the WFPs decreased due to drainage they remained adhesively attached to the soil interface, forming thin connected films of water that facilitated flow throughout the pore space. This would have biological advantages for the growing root system as the surface area available for water uptake remains high, although water quantities are reduced (Hillel, 1998). This may sustain a growing plant in short-term dry spells between rainfall events (Hunt, 2007). The total surface area of AFPs also increased with decreasing matric potential (Fig. 8; *P*<0.001). There were no significant differences between soil types (sand and clay soil) for the surface area of AFPs, but the interaction between soil category (bulk and rhizosphere soil) and matric potential was significant (*P*<0.01). Specifically, the bulk soil AFP space at 0 kPa has a much smaller total surface area (156mm^2^) compared with rhizosphere soil (373mm^2^). As the soil dried to –75 kPa, the resulting AFP space greatly increased to 354mm^2^ (56% increase) in bulk soil and to 373mm^2^ (34 % increase) in rhizosphere soil. The average surface area for AFP space was larger in the sand (0.0171mm^2^) compared with the clay (0.0168mm^2^).

### Conclusions

Here a combination of traditional and novel image-based techniques was used to investigate the effect of rhizosphere formation on soil hydraulic properties. The latter technique employed CT and image-based modelling using homogenization theory. This has the main advantage that it provides a method that can be used to derive Darcy’s law and the corresponding unsaturated hydraulic conductivity through a representative cell problem. The image-based method was also shown to capture the salient features of the WRC including the pore size and connectivity, which could be viewed and quantified in 3D across the successive drying matric potentials, therefore providing geometrical detail not possible by other methods. However, the image-based method tends to overestimate the volumetric water content at lower matric potentials, which can be attributed to possible partial volume effects and the chosen image resolution. As the matric potential is made increasingly negative, the water saturation decreases and the majority of water is trapped in smaller pores. Once these pores become comparable with or smaller than the resolution of the imaging technique, it is impossible to distinguish the difference between air and water and the method becomes less reliable. This trend is observable in both the image-based WRC and the unsaturated hydraulic conductivity. Higher resolutions are achievable by X-ray CT than used in this study, although this comes at the expense of smaller sample sizes. As this is also not desirable, a trade-off must be made between sample sizes and image resolution. Hence, a more favourable comparison between the imaging and conventional methods could be obtained through high resolution imaging of specific ROIs.

A decrease in the ability of the rhizosphere to retain water was observed; that is, the volumetric water content of the rhizosphere is lower than that of the bulk soil. When the rhizosphere forms, the hydraulic conductivity is seen to decrease significantly as the volumetric water content also decreases. This suggests that rhizosphere formation acts to reduce the soil macroporosity through densification of soil by root action, although this was soil texture dependent (Dexter, 1987; Whalley *et al.*, 2005; Aravena *et al*., 2011, 2014). This rearrangement of pore geometries by the active root system is likely to have significant implications for key processes such as water and nutrient uptake. These results provide insight into the formation of the rhizosphere in contrasting soil types. Combining this with improved numerical models which capture the dynamics of the fluid–fluid interface and advanced up-scaling techniques will provide a much more detailed picture of air and water movement in soil. The information and insights obtained on the hydraulic properties of rhizosphere and bulk soil in contrasting soil textures will enhance our understanding of rhizosphere biophysics and provide the means to improve current and future water uptake models.

## Abbreviations:

AFP: air-filled pore
CT: computed tomography
3D: three-dimensional
REV: representative elementary volume
ROI: region of interest
WFP: water-filled pore
WRC: water release characteristic.
